# Postpartum Uterine Involution in Martina Franca Jennies

**DOI:** 10.3390/ani11102762

**Published:** 2021-09-22

**Authors:** Ippolito De Amicis, Jasmine Fusi, Giuseppe Marruchella, Maria T. Zedda, Andrea Mazzatenta, Domenico Robbe, Augusto Carluccio

**Affiliations:** 1 Faculty of Veterinary Medicine, Università degli Studi di Teramo, Località Piano D’Accio, 64100 Teramo, Italy; ideamicis@unite.it (I.D.A.); gmarruchella@unite.it (G.M.); drobbe@unite.it (D.R.); acarluccio@unite.it (A.C.); 2 Department of Veterinary Medicine, Università degli Studi di Milano, Via dell’Università, 6, 26900 Lodi, Italy; 3 Department of Veterinary Medicine, Università degli Studi di Sassari, Via Vienna, 2, 07100 Sassari, Italy; zedda@uniss.it; 4 Department of Neuroscience, Imaging and Clinical Sciences, Università "d’Annunzio" di Chieti-Pescara, Via dei Vestini, 31, 66100 Chieti Scalo, Italy; amazzatenta@unite.it

**Keywords:** donkey, postpartum, uterine involution, ultrasound, histology, cytology

## Abstract

**Simple Summary:**

The assessment of a normal postpartum course is pivotal to maximize the likelihood to achieve a new pregnancy, especially in equids. However, postpartum (PP) uterine involution was studied in the horse but, to date, very little is known about the donkey, and many aspects of this fine-tuning reproductive phase remain to be clarified. The present study aimed to investigate the PP uterine involution in normal foaling Martina Franca jennies by ultrasonography, histology, and cytology. The results showed that ultrasonographic differences between the post-pregnant and non-post-pregnant uterine horn disappeared by PP Day 7. Endometrial glands area and perimeter showed a significant constant trend of decrease from PP Day 1 to PP Day 28. Epithelial thickness significantly increased from PP Days 1–3 to PP Day 7, and again, concurrently with the foal heat, to PP Day 14. Endometrial cytology showed a marked neutrophil reduction at PP Day 7, and an eosinophil increase from the first 3 days after parturition to the PP Days 7–14. Based on the combined ultrasonographic, histological, and cytological findings, the results of the present study suggest that, in Martina Franca jennies with normal foaling, the PP uterine involution can be considered complete at PP Day 14.

**Abstract:**

This study investigated the postpartum (PP) uterine involution in nine multiparous Martina Franca jennies with at term, normal, and singleton foaling. Transrectal ultrasonography performed at Days 1, 3, 7, 14, 21, and 28 showed that the uterine tip diameters did not differ between the post-pregnant (PPH) and non-post-pregnant uterine horns (NPPH), whereas the diameter of the middle PPH was larger than the NPPH until Day 7 (*p* < 0.05). The diameter of the corpora-cornual junction resulted larger in the PPH than NPPH at Day 7 (*p* < 0.05). At Day 3, the microcaruncolae were not detected. Endometrial glands (GL) number increased, with the highest value on Day 28. Endometrial GL area and perimeter decreased (*p* < 0.001) from Day 1 to Day 28. Epithelial thickness increased from Days 1–3 to Day 7 (*p* < 0.001), and concurrently with the foal heat to Day 14 (*p* < 0.001), with a decrease at Days 21–28. A marked neutrophils reduction on Day 7 and eosinophil increase from the first three days to Days 7–14 was observed. The results suggest that, in Martina Franca jennies with normal foaling, the PP uterine involution can be considered complete on Day 14.

## 1. Introduction

After parturition, the involution of the uterus and the restoration of normal genital system conditions are essential to allow a new pregnancy in all species, but even more in the horse and the donkey, in which the first postpartum estrus, known as “foal heat”, occurs shortly after parturition. This event provides a possibility for a new pregnancy, when postpartum proceeds normally, with timed uterine involution and first heat ovulation. Therefore, the assessment of a normal postpartum course is pivotal to maximize the likelihood to achieve a new pregnancy. 

In the horse, macroscopic postpartum changes can be monitored by transrectal palpation [1] and ultrasonography [2], whilst the microscopic changes can be assessed by histological [3] and cytological examinations [4], even if the latter have received little interest to monitor the postpartum period in the equine, until recently. Although ultrasonography represents the most useful and accurate tool for uterine involution monitoring in many species [5,6], histology and cytology allow a better understanding of the underlying processes leading to the involution of the uterus after parturition. The uterine involution, in fact, involves macroscopic and microscopic changes from the pregnancy-associated variations of the endometrial tissue and the other uterine wall layers [7,8].

Postpartum uterine involution was studied in the horse [9,10], but to date, very little is known about the donkey [11,12,13], and many aspects of this fine-tuning reproductive phase remain to be clarified. The donkey, in fact, was demonstrated to be similar in many reproductive aspects to the horse, however some donkey-specific studies have been reported [13,14,15,16,17,18,19] and proved to be useful for a more correct management of reproduction in this species. According to the data updated in the year 2019, the Martina Franca donkey breed, with a consistency of 107 approved for breeding jackasses and 265 for jennies, is one of the Italian donkey breeds with requested programs for population conservation and number increase. Where endangered populations are concerned, the development of preservation programs based on scientific knowledge is recommended. Among the reproductive aspects that need to be better investigated, the postpartum uterine involution remains not fully investigated in donkeys.

Therefore, for a better management of reproduction in an endangered population, the present study aimed to investigate the postpartum (PP) uterine involution in Martina Franca jennies by ultrasonography, histology, and cytology, with the purpose to identify the timing for the complete uterine involution. 

## 2. Materials and Methods

### 2.1. Ethics

The project was approved by the Committee on Animal Research and Ethics of the Universities of Chieti-Pescara and Teramo (http://www.unich.it/unichieti/appmanager/federati/CEISA), Protocol #45/2013/CEISA/COM, approval date 16 July 2013.

### 2.2. Animals

The study was performed on 9 multiparous (2–4 parity) Martina Franca jennies, aged 6–12 (mean ± SD: 9.4 ± 3.1) years old, 340–380 (mean ± SD: 357 ± 27.0) kg body weight, housed at the Veterinary Teaching Farm of the University of Teramo, Italy, and fed daily with standard hay ad libitum and commercial equine fodder (4 kg)*,* in a single breeding season from March to May 2020.

The jennies were healthy, regularly vaccinated, and dewormed, and were monitored from the time of mating, throughout pregnancy, until parturition, by routine clinical and ultrasonographic examinations to assess the general health, normal pregnancy course, and fetal development and well-being. The body condition score at foaling ranged between 3/5 and 4/5.

At approaching parturition, detected by udder enlargement, jennies were moved from the open paddocks to individual boxes provided with a close-circuit television system for foaling surveillance. According to a previous study [15], foaling was considered as normal only when singleton, at term, unassisted deliveries, giving birth to healthy, mature, and viable [20] donkey foals, and normal placental expulsion, occurred. 

### 2.3. Postpartum Uterine Involution, Endometrial Biopsies, and Foal Heat

The PP was considered normal when the jennies did not show, or showed only little amounts of lochia, absence of systemic signs of inflammation, and did not require medical treatments [4]. Only clinically healthy jennies with negative bacteriologic uterine samples were enrolled.

Transrectal ultrasonography was performed, always by the same operator, the day after foaling (PP Day 1), and again at PP Days 3, 7, 14, 21, and 28, using an ultrasound machine (Mindray 2200 Vet, Mindray Medical Italy Srl, Trezzano sul Naviglio, Milan, Italy) mounting a linear 5–10 MHz transducer. 

The cross-sectional diameter was measured at the post-pregnant (PPH) and non-post-pregnant uterine horns (NPPH) at the level of the tip (T), middle (M), and corpora-cornual junction (CCJ) regions. For each section, the diameter was measured three times and the median value was considered as the final value. The ovaries were also scanned to assess the first PP ovulation. 

At the same times of ultrasound examination, endometrial biopsies were also performed at the base of the uterine horn, using Jackson equine alligator uterine biopsy forceps (Jorgensen Lab Inc, Loveland, CO, USA) to collect a specimen of at least 4 × 28 mm, stored in a 4% buffered formalin solution. Sections of 5 µm were routinely processed and stained with hematoxylin and eosin [21].

Daily teasing from the third day after parturition was performed, followed by the ultrasonographic monitoring of dominant follicle development.

Therefore, the intervals between parturition and the beginning of foal heat, between parturition and the day of the first PP ovulation, and the diameter of the ovulating follicle were recorded.

None of the jennies were voluntarily inseminated at the foal heat.

### 2.4. Endometrial Histology and Cytology

Biopsies were used for the computerized histological evaluation (ImageJ 1.50i Wayne Rasband, National Institutes of Health, Bethesda, MA, USA) of epithelial thickness (ET), measured 3 times in each field view, to evaluate the microcaruncolar (MC) presence, perimeter, area, and characteristics, and to assess the endometrial glandular (GL) number, perimeter, and area. For MC, GL, and ET, at least 10 fields were observed by light microscope (Nikon Ecilpse E600, Nital SpA, Moncalieri, Turin, Italy), at 10× magnification for MC and GL assessment, but at 20× magnification for the ET evaluation. All images were digitally acquired by a Nikon Digital camera DXM 1200 (Nital SpA, Moncalieri, Turin, Italy) mounted on a microscope through the software Nikon ACT-1 Version 2.20 (Copyright © 2000, Nikon Corporation (Nital SpA, Moncalieri, Turin, Italy)).

Neutrophil, eosinophil, and mononucleate cells’ amount evaluation was performed by light microscope (Nikon Ecilpse E600, Nital SpA, Moncalieri, Turin, Italy), observing at least 10 fields at 20× magnifications. For each cell type (neutrophil, eosinophil, mononucleate), the cells’ amount was defined by a score from 0 to 4, in which score 0 corresponds to complete absence of cells in the objective fields, score 1 refers to cells’ amount ≤ 25%, score 2 to cells’ amount of 26–50%, score 3 to cells’ amount of 51–75%, and score 4 to cells’ amount of 76–100%.

### 2.5. Statistical Analysis

Possible differences in uterine ultrasonographic measurements between all the PPH and NPPH sections at all sampling times were statistically evaluated by ANOVA for repeated measures, followed by the Bonferroni post hoc test. Possible differences in PPH histological and cytological findings among all sampling times were assessed by ANOVA for repeated measures, followed by the Bonferroni post hoc test for histological findings. Since cytological data were not normally distributed, the non-parametric Kruskal–Wallis test was used.

The uterine involution was considered as the first sampling time in which the ultrasonographic findings between the PPH and NPPH did not show significant differences, and the histological and cytological findings within the PPH showed no significant differences in comparison to the previous sampling time.

All data are expressed as mean ± SD and statistical significance was set at *p* < 0.05 (Jamovi, ver. 1.2).

## 3. Results

### 3.1. Clinical Findings 

After a normal singleton gestation course length of 355 ± 16 days, the 9 jennies gave birth, unassisted, by spontaneous vaginal delivery to 9 mature, healthy, and viable foals, 5 females (birthweight: 34 ± 6 Kg) and 4 males (birthweight: 33.3 ± 2 Kg), and displayed normal placental expulsion and negative uterine bacteriologic examination, fulfilling the inclusion criteria.

After delivery, all the jennies displayed a normal PP, with foal heat beginning on 6.5 ± 0.9 days PP. 

### 3.2. Ultrasonography

**Figure 1:**
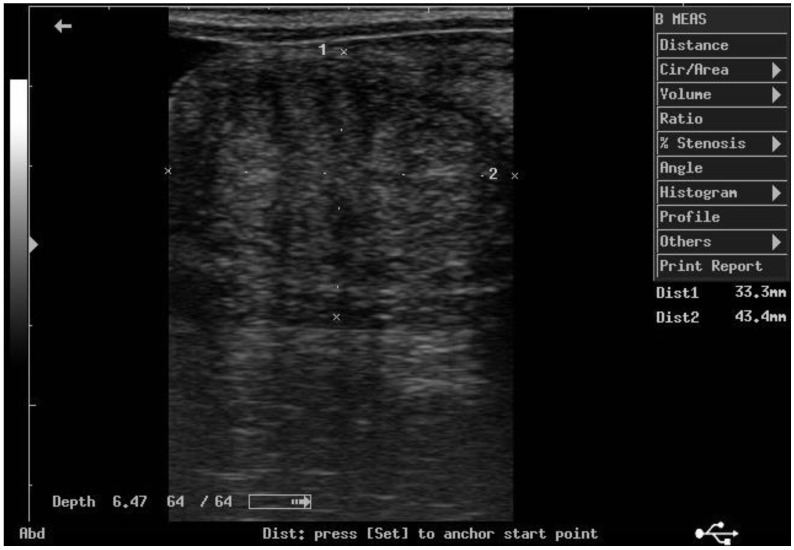
Ultrasound image of the middle tract of the post-pregnant uterine horn, at day 7 postpartum.

**Figure 2:**
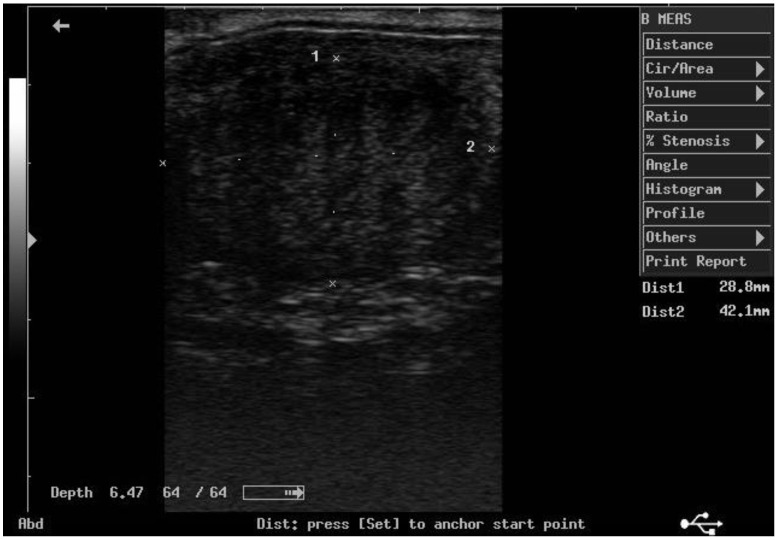
Ultrasound image of the middle tract of the non-post-pregnant uterine horn, at day 7 postpartum.

**Figure 3:**
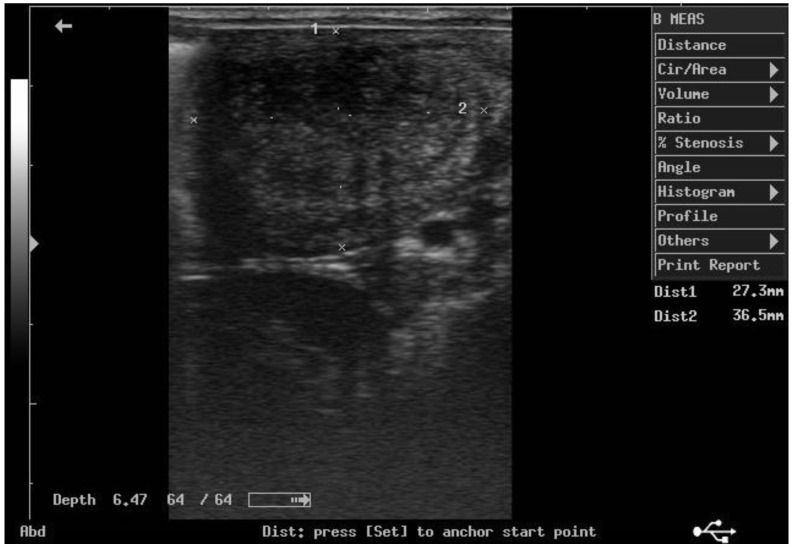
Ultrasound image of the middle tract of the post-pregnant uterine horn, at day 14 postpartum.

**Figure 4:**
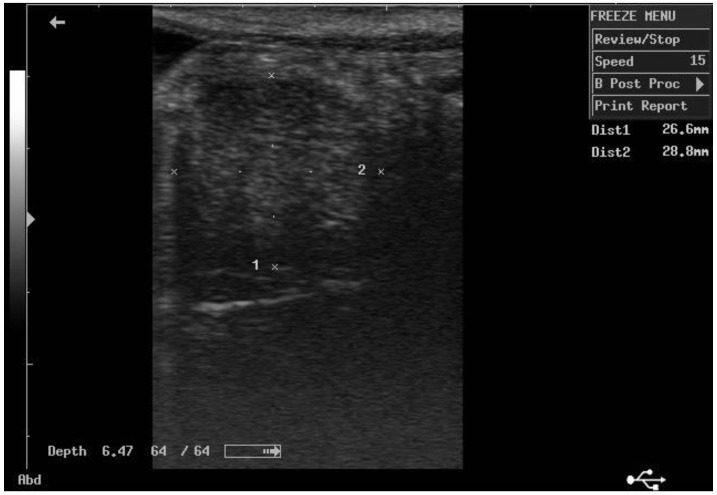
Ultrasound image of the middle tract of the non-post-pregnant uterine horn, at day 14 postpartum.

According to ultrasonographic records (Table 1), the mean T diameters did not differ significantly between PPH and NPPH at any time, whereas the mean M diameter of the PPH was larger than the NPPH only until PP Day 7 (Figure 1, Figure 2, Figure 3 and Figure 4). The Bonferroni post hoc test confirmed the ultrasonographic data obtained by ANOVA for repeated measures, when PPH and NPPH were considered across time. Moreover, when only time was assessed, the Bonferroni post hoc test highlighted similar significant changes occurring between PP Days 1–3 and 14–28 (*p*-value ranging between <0.05 and <0.001) and between PP Days 7 and 14–28 (*p*-value ranging between <0.05 and <0.01) for the M and CCJ. In relation to T, significant changes were detected among all the times of observation, with *p*-value ranging between <0.05 and <0.001.

The diameter of the CCJ in the PPH was not measurable until PP Day 3, then the mean diameter of the CCJ resulted larger in the PPH in comparison to NPPH only at PP Day 7. The first PP ovulation, detected by ultrasonography, occurred 11.9 ± 1.3 days after foaling. At the first PP ovulation, the follicle diameter was 45.1 ± 2.8 mm.

### 3.3. Histology

**Figure 5:**
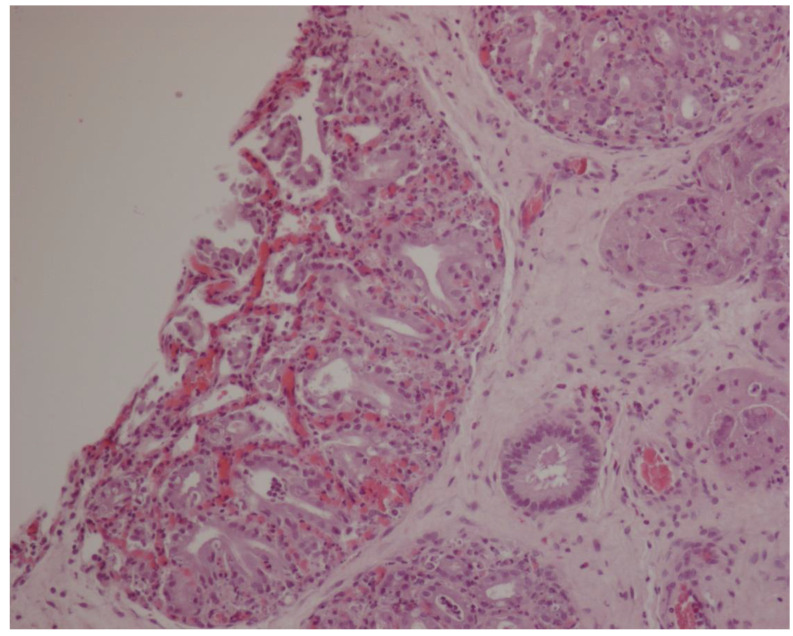
Histologic sampling at day 1 postpartum. In the middle of the figure, a microcaruncle is visible (HE-10× magnification).

Histology showed that MC were detectable only on PP Day 1 (Figure 5), when perimeter and area measured 40,731 ± 13,651 μm and 130,787 ± 76,562 μm^2^, respectively. At PP Day 3, MC were not detected anymore, whilst clear signs of cells’ karyorrhexis and cytoplasmatic vacuolization were observed, providing indications of involution.

**Figure 6:**
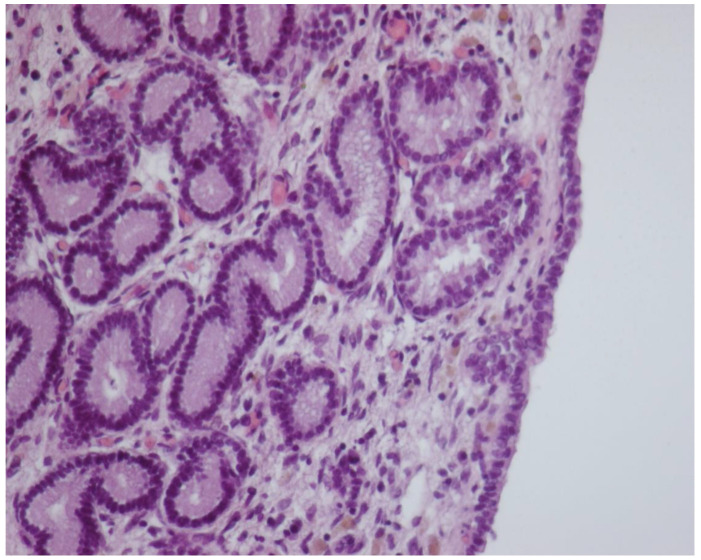
Histologic sampling at postpartum day 14. Cubic continuing epithelium with numerous and well-developed glandular structures (HE-20× magnification).

In comparison to PP Days 1–7, GL mean number showed a significant trend of increase from PP Day 14 (Figure 6), reaching the highest value on PP Day 28.

Perimeters of GL and GL area showed a significant constant trend of decrease from PP Day 1 to Day 28, failing to provide a suitable cutoff time for uterine involution. The mean ET significantly increased from PP Day 1 to Days 3–7, with a further increase, concurrently with the foal heat, at PP Day 14, followed by a decrease at PP Days 21–28, returning to a similar thickness as at PP Day 3 (Table 2). The Bonferroni post hoc test confirmed the histological data obtained by ANOVA for repeated measures.

### 3.4. Cytology

The number of neutrophil cells, evaluated by a score system, showed a significantly lower score between PP Day 1 and PP Days 7–28, between PP Day 3 and PP Days 7–28, and between PP Days 7–21 and PP Day 28 (Table 3).

The amount of eosinophil cells, evaluated by a score system, showed a higher significant mean score between PP Day 1 and PP Days 7–14, between PP Day 3 and PP Days 7–14, and a lower significant mean score between PP Day 7 and PP Day 21 (Table 3). Mononucleate cells were found only seldom, as small aggregates, and limited to PP Days 7–14.

## 4. Discussion

Uterine involution, a complex process involving anatomical, histological, and cytological changes, is pivotal to allow a new gestation. Therefore, its correct comprehension is needed for the best management of postpartum in all species, and even more in equids, in which the first postpartum heat occurs soon after parturition. Moreover, in programs for population increase, such as the endangered Martina Franca donkey breed, maximizing the pregnancy achievement plays a crucial role. Although ultrasonography is known to be the most common tool for monitoring the PP uterine involution, studies of histological and cytological changes are needed to understand the mechanisms underlying the uterine involution, providing more precise information for the better management of reproduction. In comparison to the horse mare, only few previous studies [11,12,13] investigated some aspects of uterine involution in the donkey, mainly focusing on foal heat characteristics, but the overall picture of ultrasonographical, histological, and cytological changes was lacking.

Ultrasonography, per se, does not provide precise information about the full functionality of an organ, but remains the most common tool for the management of equine female reproduction, providing immediate evaluation of ovarian and uterine structures and characteristics, especially suitable under field conditions.

In the present study, transrectal ultrasonography performed on PP Days 1–3 did not allow to obtain adequate pictures for correctly measuring the cross-sectional diameter of the CCJ regions of the PPH, differently to a previous study [11] in which measurements were performed already at Day 3 after foaling.

Altogether, the ultrasonographic findings showed that T diameters did not differ significantly between PPH and NPPH at any time, whereas the differences between the mean M and CCJ diameters of the PPH and the NPPH disappeared after PP Day 7, suggesting that uterine involution assessed by ultrasonography could be considered completed by the subsequent observation, performed on PP Day 14. These findings disagree with previous data from the same donkey breed [13], in which, in most jennies, the recovery of the normal uterine diameter was detected by PP Day 21. Even in French donkeys [11], the mean time for uterine involution was about 22 days. Those differences could be attributable to the modality and frequency of ultrasonographic monitoring (equipment, frequency, etc.) and maybe also to the different donkey breed enrolled in the study [11]. The results of the present study are more similar with data reported for the horse mare, in which by PP Days 15–16, an adequate degree of uterine involution was detectable [9,10].

Uterine biopsies showed that MC disappeared soon after foaling and they were not detectable by PP Day 3, while in pony mares, Jischa and colleagues did not find microcaruncles in specimens collected for biopsy by Day 9 [10]. Endometrial GL number increased significantly from PP Day 14, while perimeter and area decreased constantly from foaling to PP Day 28. Similarly, in pony mares, endometrial glands are reported to be less dilated at Days 9 and 16 as compared to Day 1 [10]. These findings seem to suggest that the secretive function of the endometrium is also restored as early as 14 days after foaling in Martina Franca jennies. In the mare, the glandular density was reported to show a trend of increase during the first 2 weeks after foaling [3,22]. In the present study, the ET was significantly increased at PP Day 7, further increasing at PP Day 14, concurrently with the foal heat. This agrees with the recognized increase of the height of the epithelium during estrus in the mare [8]. During postpartum, the height of the epithelium was reported to increase in the mare 5 days after foaling [23], very similar to the finding of the present study in jennies.

The assessment of eosinophil and neutrophil cells’ amount in the PPH, performed by a score system, showed a marked neutrophils reduction, of about one half–one third of the initial value, at PP Day 7, maybe indicating that, as early as Day 7 PP, in normal foaling Martina Franca jennies, the uterine mucosa is already regenerated, as recently reported in the mare [4]. In mares, in fact, the luminal epithelium was reported to become intact by Days 4–7 PP [9,24]. Moreover, in mares, the reduction of neutrophil cells in the endometrium was reported to be an indicator of undisturbed puerperal development [4]. In pony mares, this process is slightly postponed at Day 9, at which time neutrophils had largely ceased to be seen, substituted by lymphocytes and macrophages [10].

A different trend was observed for eosinophils, showing a significant, more than doubled, increase from the first 3 days after parturition to the PP Days 7–14, followed by a decrease to values similar to those recorded during the first 3 days after foaling. Eosinophils were reported to be more common on Day 5 PP in the mare [23]. The presence of eosinophils could be supposed to represent an immune response in the early postpartum period, as previously suggested for the initial interaction between the conceptus and the uterus in the mare [25]. The seldom observation of small mononucleate cell aggregates, only at PP Days 7–14, seems to suggest a minor role of these cells in the process of uterine involution in jennies.

In the present study, all the jennies showed the onset of foal heat on PP Day 6.5 ± 0.9, very similar to the PP Day 7.4 ± 2.1 previously reported for the same donkey breed [13]. In the French donkeys [11], a significant delay in uterine involution was reported for those jennies in which the foal heat occurred earlier than Day 9 after foaling, possibly because of the different donkey breed enrolled. In the present study, the evaluation of a similar possible effect on PP uterine involution was not possible, because of the small number of studied animals, and because in all the jennies, the foal heat occurred homogeneously earlier than PP Day 9. However, because uterine involution was completed in all jennies by PP Day 14, it is possible to suggest that the early occurrence of foal heat does not seem to influence the process of uterine involution in Martina Franca donkeys.

## 5. Conclusions

In conclusion, the present study, thanks to the combined results of ultrasonographical, histological, and cytological findings, provided a more complete evidence for the timing of PP uterine involution in normal foaling Martina Franca jennies.

Taking all the findings together, the present study showed that, in the normal foaling Martina Franca jennies investigated, the timing of PP uterine involution was 14 Days PP, similar to the results reported for the horse mare, and a bit quicker than data previously reported for another donkey breed. Besides the usefulness of ultrasonography under practical settings, in Martina Franca jennies, endometrial histology and cytology were also shown to be valuable tools for a more precise evaluation of uterine involution progression, as recently reported for the mare [4].

## Figures and Tables

**Table 1:** Postpartum cross-sectional diameter measured (mean ± SD) at the non-post-pregnant and post-pregnant uterine horns at the level of the tip, middle, and corpora-cornual junction regions, at the different sampling times, in the 9 Martina Franca jennies.

	Cross-Sectional Diameter (mm) Non-Post-Pregnant Horn			Cross-Sectional Diameter (mm) Post-Pregnant Horn		
PP Day	Tip	Middle	Corpora-Cornual Junction	Tip	Middle	Corpora-Cornual Junction
1	41.8 ± 1.2	59.1 ± 0.8 ^A^	86.0 ± 1.2	44.0 ± 1.3	76.0 ± 0.8 ^B^	Not assessable
3	38.3 ± 1.8	56.7 ± 1.1 ^A^	84.5 ± 2.0	41.0 ± 0.8	71.6 ± 0.7 ^B^	Not assessable
7	35.5 ± 0.9	54.0 ± 1.4 ^A^	77.8 ± 1.4 ^a^	37.3 ± 0.8	62.6 ± 0.7 ^B^	96.8 ± 1.5 ^b^
14	29.0 ± 1.3	46.8 ± 1.5	67.8 ± 1.6	30.5 ± 1.1	47.1 ± 2.2	72.3 ± 2.4
21	26.6 ± 0.6	43.3 ± 1.3	64.6 ± 1.0	25.7 ± 1.0	45.2 ± 1.3	64.3 ± 1.4
28	24.5 ± 0.6	43.3 ± 0.8	58.1 ± 1.1	24.8 ± 0.7	42.0 ± 1.2	59.6 ± 1.4

^A,B^*p* < 0.05 and ^a,b^ *p* < 0.05 denote within row statistical significance.

**Table 2:** Postpartum endometrial glandular number, perimeter, area, and epithelium thickness (mean ± SD) in the different sampling times in the 9 Martina Franca jennies.

PP Day	Number	Perimeter (mm)	Area (mm^2^)	Epithelium Thickness (mm)
1	129 ± 110.8 ^♦^^§^^♣^	8.8 ± 3.4 ^A^	5.3 ± 4.2 ^AA^	1.0 ± 0.3 ^A^
3	173 ± 124.6 ^♠^^•^	8.6 ± 3.6 ^A^	5.2 ± 4.2 ^AA^	1.3 ± 0.4 ^B^
7	240 ± 127.0 ^♥^^#^	7.4 ± 3.2 ^B^	3.9 ± 3.1 ^BB^	1.3 ± 0.4 ^B^
14	396 ± 320.8 ^♦^	7.2 ± 2.9 ^B^	3.6 ± 2.5 ^BB^	1.4 ± 0.4 ^C^
21	519 ± 220.0 ^♥^^§^^♠^	6.5 ± 2.1 ^C^	2.8 ± 2.0 ^CC^	1.2 ± 0.3 ^B^
28	546 ± 240.5 ^#^^♣^^•^	6.2 ± 2.9 ^C^	2.5 ± 2.2 ^CC^	1.2 ± 0.3 ^B^

^A,B,C^ Denote within column significant differences with *p* < 0.001. ^AA,BB,CC^ Denote within column significant differences with *p* < 0.001. Glandular number ^♦^ *p* < 0.05; ^♥^ *p* < 0.01; ^#^ *p* < 0.01; ^§^ *p* < 0.001; ^♣^ *p* < 0.001; ^♠^ *p* < 0.001; ^•^ *p* < 0.001.

**Table 3:** Score (mean ± SD) for postpartum eosinophil and neutrophil cells’ amount evaluation at the different sampling times, in the 9 Martina Franca jennies.

	PP Day					
	PP Day 1	PP Day 3	PP Day 7	PP Day 14	PP Day 21	PP Day 28
Eosinophil Score	0.51 ± 0.42 ^§#^	0.73 ± 0.49 ^♥^^♣^	1.94 ± 0.82 ^§^^♥^^♦^	1.57 ± 0.82 ^#^^♣^	0.96 ± 0.87 ^♦^	0.94 ± 1.20
Neutrophil Score	1.16 ± 0.46 ^§#^^♥^^♦^	1.51 ± 0.69 ^♣^^♠^^AB^	0.51 ± 0.30 ^§^^♣^^C^	0.52 ± 0.34 ^#^^♠^^D^	0.37 ± 0.13 ^♥^^AE^	0.17 ± 0.16 ^♦^^BCDE^

Eosinophils: **^§^** *p* < 0.001; ^#^ *p* < 0.01; ^♥^ *p* < 0.01; ^♣^ *p* < 0.05; ^♦^ *p* < 0.05. Neutrophils: ^§^ *p* < 0.01; **^#^** *p* < 0.01; ^♥^ *p* < 0.001; ^♦^ *p* < 0.001; ^♣^ *p* < 0.01; ^♠^ *p* < 0.01; ^A^ *p* < 0.001; ^B^ *p* < 0.001; ^C^ *p* < 0.01; ^D^ *p* < 0.05; ^E^ *p* < 0.05.

## Data Availability

Data are available upon reasonable request to the authors.
